# Origin of an Alternative Genetic Code in the Extremely Small and GC–Rich Genome of a Bacterial Symbiont

**DOI:** 10.1371/journal.pgen.1000565

**Published:** 2009-07-17

**Authors:** John P. McCutcheon, Bradon R. McDonald, Nancy A. Moran

**Affiliations:** 1 Center for Insect Science, University of Arizona, Tucson, Arizona, United States of America; 2 Department of Ecology and Evolutionary Biology, University of Arizona, Tucson, Arizona, United States of America; Université Paris Descartes, INSERM U571, France

## Abstract

The genetic code relates nucleotide sequence to amino acid sequence and is shared across all organisms, with the rare exceptions of lineages in which one or a few codons have acquired novel assignments. Recoding of UGA from stop to tryptophan has evolved independently in certain reduced bacterial genomes, including those of the mycoplasmas and some mitochondria. Small genomes typically exhibit low guanine plus cytosine (GC) content, and this bias in base composition has been proposed to drive UGA Stop to Tryptophan (Stop→Trp) recoding. Using a combination of genome sequencing and high-throughput proteomics, we show that an α-Proteobacterial symbiont of cicadas has the unprecedented combination of an extremely small genome (144 kb), a GC–biased base composition (58.4%), and a coding reassignment of UGA Stop→Trp. Although it is not clear why this tiny genome lacks the low GC content typical of other small bacterial genomes, these observations support a role of genome reduction rather than base composition as a driver of codon reassignment.

## Author Summary

The genetic code, which relates DNA sequence to protein sequence, is nearly universal across all life. Examples of recodings do exist, but new instances are rare. Genomes that exhibit recodings typically have other extreme properties, including reduced size, reduced gene sets, and low guanine plus cytosine (GC) content. The most common recoding event, the reassignment of UGA to Tryptophan instead of Stop (Stop→Trp), was previously known from several mitochondrial and one bacterial lineage, and it was proposed to be driven by extinction of the UGA codon due to reduction in GC content. Here we present an unusual bacterial genome from a symbiont of cicadas. It exhibits the UGA Stop→Trp reassignment, but has a high GC content, showing that reduction in GC content is not a necessary condition for this recoding. This symbiont genome is also the smallest known for any cellular organism. We therefore propose gene loss during genome reduction as the common force driving this code change in bacteria and organelles. Additionally, the extremely small size of the genome further obscures the once-clear distinction between organelle and autonomous bacterial life.

## Introduction

**Figure 1:**
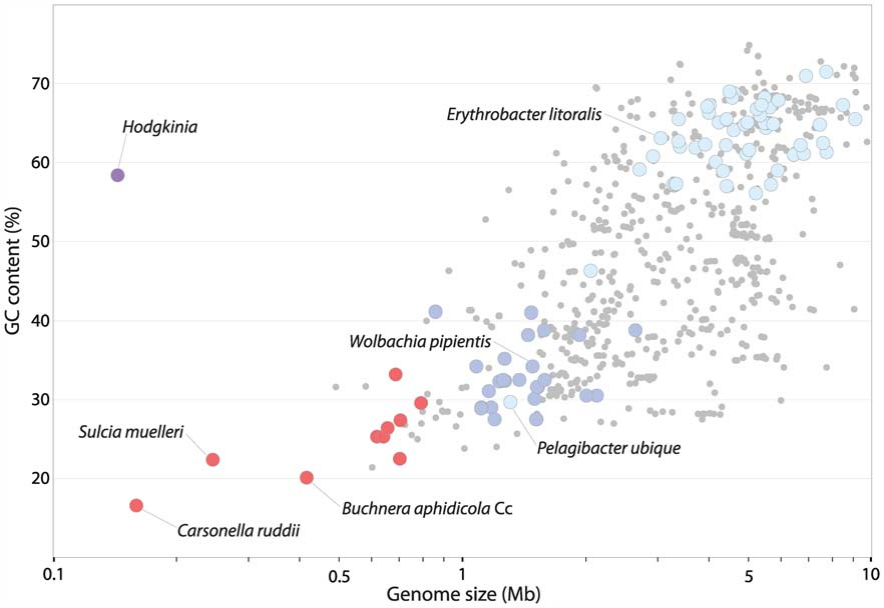
Relationship between genome size and GC content for sequenced Bacterial and Archaeal genomes. Obligately intracellular insect symbionts are shown as red circles, obligately intracellular α-Proteobacteria as dark blue circles, *Hodgkinia* as a purple circle (as it is both an obligately intracellular α-Proteobacteria and an insect symbiont), and all other α-Proteobacteria as light blue circles. Most other Bacteria and Archaea are represented by small gray circles, although some have been removed for clarity, and the plot is truncated at 10 Mb.

The GC content of bacterial genomes has been known to vary widely since at least the 1950s [1]. Currently sequenced genomes range from 17–75% GC and show a strong correlation between genome size and GC content [2]–[4] (Figure 1). The tiny genomes of symbionts of sap-feeding insects are extreme exemplars of this relationship: *Carsonella ruddii* [5], *Sulcia muelleri* [6], and *Buchnera aphidicola* Cc [7], which represent three independently evolved endosymbiont lineages, have the smallest and most GC-poor genomes yet reported (Figure 1). These bacteria have a strict intracellular lifestyle, and this shift from a free-living state to an obligate intracellular one greatly reduces the effective population size of the bacteria, in part by exposing them to frequent population bottlenecks as they are maternally transmitted during the insect lifecycle [2],[3],[8]. This population structure leads to an increase in genetic drift, and this increase, combined with the constant availability of the rich metabolite pool of the insect host cell, is thought to explain the massive gene loss and high rate of sequence evolution seen in intracellular bacteria [2],[3]. Sequence evolution is also likely accelerated by an increased mutation rate, stemming from the loss of genes involved in DNA repair during genome reduction [4]. This loss of repair enzymes may contribute to the AT bias of small bacterial genomes since common chemical changes in DNA, cytosine deaminations and guanosine oxidations, both lead to mutations in which an AT pair replaces a GC pair, if left unrepaired [9],[10]. Indeed, the properties of all symbiont genomes published to date fit well within this framework (Figure 1).

The UGA Stop→Trp recoding, found in the mycoplasmas and several mitochondrial lineages, is associated with both genome reduction and low GC content [11]–[13]. Under the “codon capture” model, a codon falls to low frequency and is then free to be reassigned without major fitness repercussions. Applying this model to the UGA Stop→Trp recoding, mutational bias towards AT causes each UGA to mutate to the synonym UAA without affecting protein length [14],[15]. When the UGA codon subsequently reappears through mutation, it is then free to code for an amino acid [14],[15]. While some have argued that codon capture is insufficient to explain many recoding events [11],[12], the fact that all known UGA Stop→Trp recodings have taken place in high AT genomes [11],[16] makes the argument attractive for this recoding.

Here we describe the genomic properties of an α-Proteobacterial symbiont (for which we propose the name *Candidatus* Hodgkinia cicadicola) from the cicada *Diceroprocta semicincta* (Davis 1928) [17]. We show that at only 143,795 bps it has the smallest known cellular genome, but has a high GC content of 58.4% and a recoding of UGA Stop→Trp. We hypothesize that gene loss associated with genome reduction is a critical step in this recoding, rather than mutational pressure favoring AT. Specifically, we suggest that loss of translational release factor RF2, which recognizes the UGA stop, was the unifying force driving the recoding in *Hodgkinia* as well as in certain other small AT-rich genomes.

## Results

**Figure 2:**
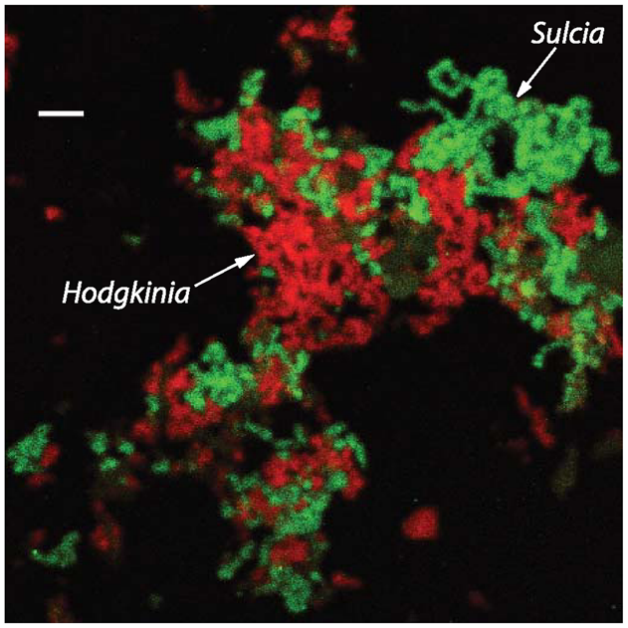
*Sulcia* (green) and *Hodgkinia* (red) both have large tubular cell morphologies and are closely associated within the same bacteriocytes. Scale bar is 10 µm.

Previous work revealed that some cicadas had *Sulcia* as symbionts [18], but the identity of other symbionts, if any, was unknown. To identify any coexisting symbionts, we amplified and sequenced 16S rRNA genes from cicada bacteriomes (organs containing symbiotic bacteria). A second bacterial type was discovered and found to have large and irregularly shaped cells (Figure 2). Unusual cell morphologies have been observed in other bacteria with tiny genomes [5],[18], suggesting that this symbiont species might also have a small genome. Preliminary analysis using the Naive Bayesian rRNA Classifier [19] at the Ribosomal Database Project website [20] placed the new 16S rDNA sequence in the α-Proteobacteria with 100% confidence and, more specifically, within the Rhizobiales with 86% confidence. Because all other endosymbiotic α-Proteobacteria with small genomes are members of the Rickettsiales (e.g. *Wolbachia*, *Rickettsia*, and *Erhlichia*), we were interested in obtaining genomic data to further characterize this seemingly strange bacterium.

Genome sequencing revealed that *Hodgkinia* had some properties that were similar to other endosymbiont genomes, such as high coding density and shortened open reading frames (Table 1). But other aspects of the *Hodgkinia* genome suggested a highly atypical bacterial genome structure. In particular, the genome was only 144 kb, and thus even smaller than other known symbiont genomes, but had an unusually high GC content of about 58%. To our knowledge, this is an unprecedented combination of genome size and base composition (Figure 1). Additionally, initial rounds of gene prediction revealed that many protein-coding regions were interrupted by putative stop codons. Our previous experience [6] suggested that this could be due to errors in homopolymeric run lengths predicted by Roche/454 sequencing technology. However, the addition of Illumina/Solexa data indicated that the interrupted reading frames were not caused by sequencing errors. We noticed that computational translation of the genome with the NCBI genetic code 4 (UGA Stop→Trp) afforded full-length protein sequences, which immediately suggested that *Hodgkinia* might use an alternative genetic code.

**Table 1:** Genomic properties of representative bacteria within phyla containing species with both large and highly reduced genomes.

	γ-Proteobacteria			α-Proteobacteria			Bacteroidetes		
	*Escherichia coli* K12	*Buchnera aphidicola* Cc	*Carsonella ruddii* PV	*Rhizobium etli* CFN 42	*Pelagibacter ubique* HTCC1062	*Hodgkinia cicadicola*	*Bacteroides thetaiotaomicron* VPI-5482	*Amoebophilus asiaticus* 5a2	*Sulcia muelleri* GWSS
Genome Size (bp)	4,639,675	422,434	159,662	4,381,608	1,308,759	143,795	6,260,361	1,884,364	245,530
G+C %	50.8	20.1	16.6	61.0	29.7	58.4	42.8	35.0	22.4
Number of genes	4418	362	213	4126	1389	189	4864	1494	263
Coding density	88.5	87.7	97.3	87.3	96.1	95.1	89.9	84.1	96.0
Average CDS length	950.1	995.7	825.9	936.5	925.8	776.8	1173.5	1134.9	996.3

Protein-coding (CDS), tRNA, and rRNA genes were included in the number of genes and coding density calculations. *Hodgkinia*, *C. ruddii*, and *S. muelleri* are the three smallest cellular genomes known; all are insect symbionts.

**Figure 3:**
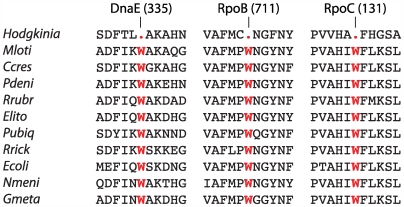
Conserved positions encoded by UGA in *Hodgkinia* correspond to tryptophan (W) in other Proteobacteria. *M. loti* (*Mloti*), *C. crescentus* (*Ccres*), *P. denitricans* (*Pdeni*), *R. rubrum* (*Rrubr*), *E. litoralis* (*Elito*), *P. ubique* (*Pubiq*), and *R. rickettsii* (*Rrick*) are all α-Proteobacteria; *E. coli* (*Ecoli*), γ-Proteobacteria; *N. meningitidis* (*Nmeni*), β-Proteobacteria; and *G. metallireducens* (*Gmeta*), δ-Proteobacteria. Partial sequences from the proteins DnaE (DNA polymerase III, α subunit), RpoB (RNA polymerase, β subunit), and RpoC (RNA polymerase, β′ subunit) are shown; the positions indicated at the top of the alignments are from the *Hodgkinia* proteins.

Analysis of the gene complement of *Hodgkinia* revealed that the genome contains a homolog of *prfA*, encoding translational Release Factor RF1, which recognizes the stop codons UAA and UAG, but does not contain a homolog of *prfB* (RF2), which recognizes UAA and UGA. RF2 is dispensable if UGA is not used as a stop codon, and the loss of RF2 combined with recoding of UGA Stop→Trp is known in *Mycoplasma* species [13],[21],[22]. Additionally, the anticodon of the sole tRNA-Trp gene in *Hodgkinia* (*trnW*) has mutated from CCA to UCA, which allows recognition of both the normal tryptophan codon (UGG) and the putatively recoded UGA stop codon under Crick's wobble rules for codon-anticodon pairing [23]. This tRNA-Trp mutation has also been observed in mitochondrial genomes that have the UGA Stop→Trp recoding [24]. Additionally, it was observed that UGA codons in *Hodgkinia* open reading frames correspond to the position of conserved tryptophan residues in homologous proteins of other bacteria (Figure 3). Cumulatively, these data strongly suggested that UGA encodes tryptophan in *Hodgkinia*.

**Figure 4:**
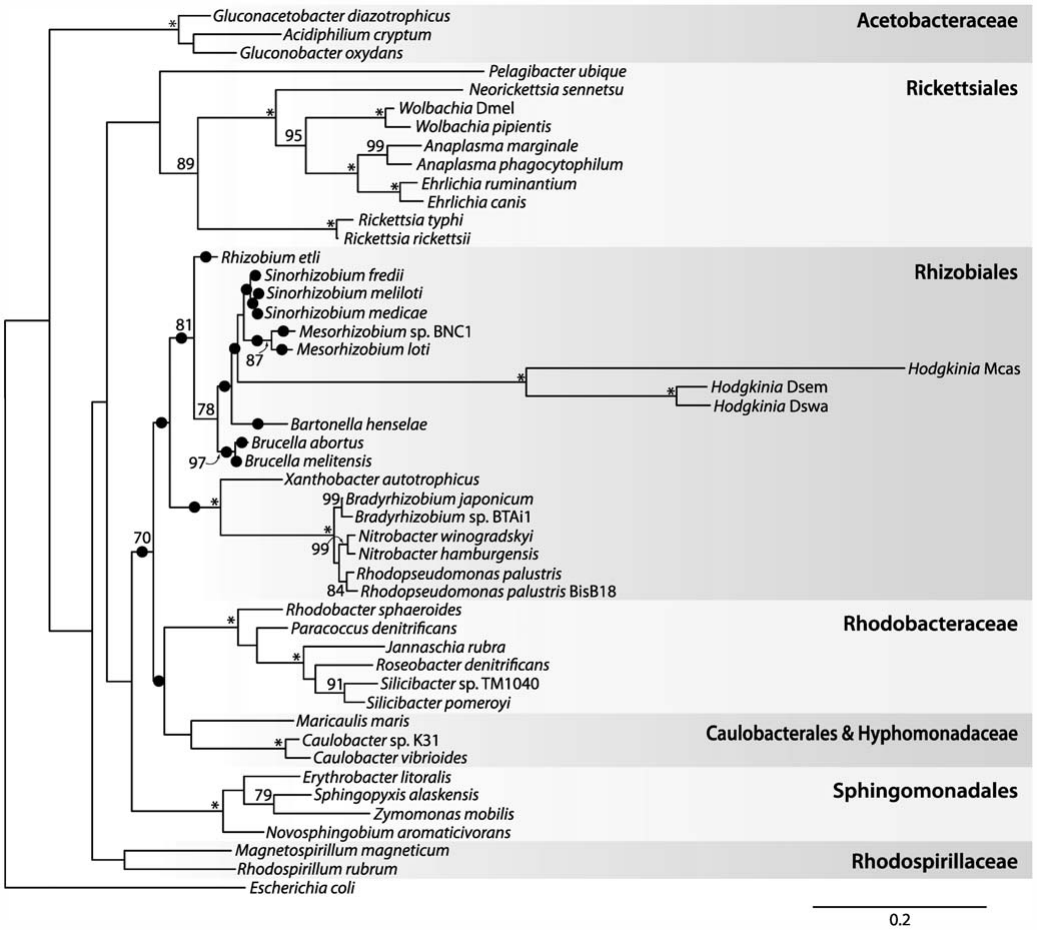
Relationship of *Hodgkinia* to other α-Proteobacteria based on small subunit ribosomal DNA sequences. By itself, this maximum likelihood tree gives moderate support (81/100 bootstrap trees) for the grouping of *Hodgkinia* with the Rhizobiales. The twenty highest scoring positions for the *Hodgkinia* clade under a non-homogenous GC content model are indicated with black circles, and provide additional support for *Hodgkinia*'s grouping in the Rhizobiales. Abbreviations are Mcas, *Magicicada cassini*; Dswa, *Diceroprocta swalei*; and Dsem, *Diceroprocta semicincta*. Asterisks indicate 100% bootstrap support; values less than 70% are not shown. Scale bar denotes substitutions per site.

**Figure 5:**
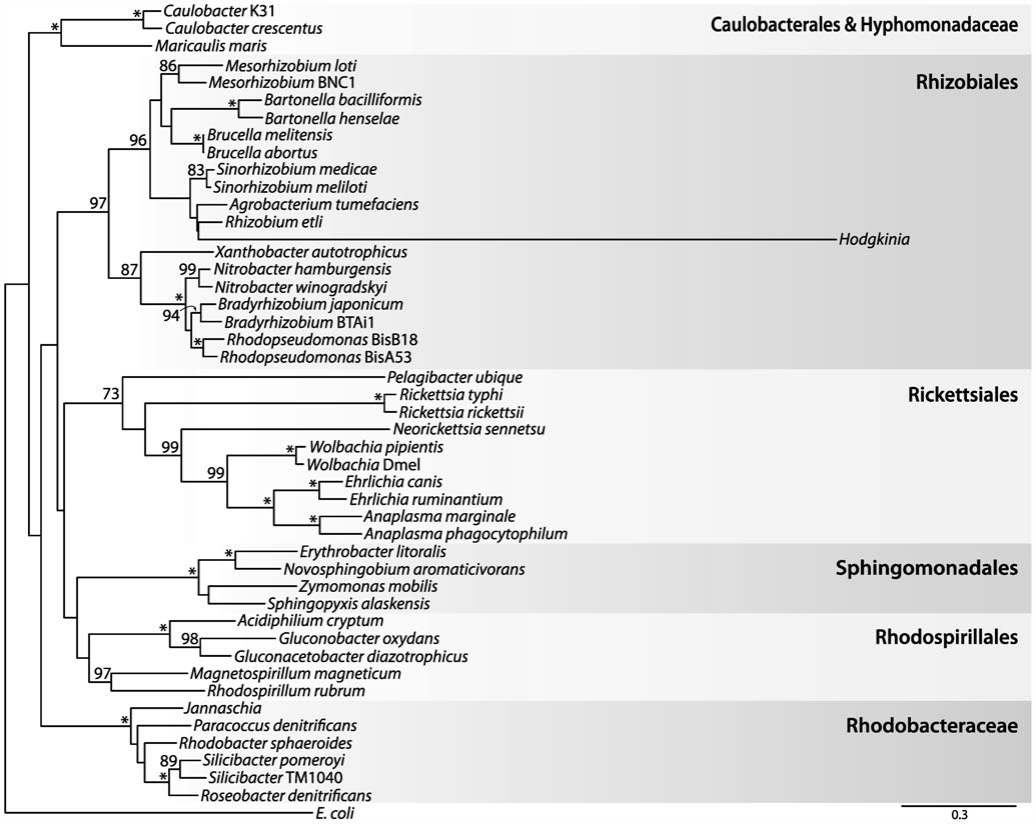
Relationship of *Hodgkinia* to other α-Proteobacteria based on protein sequences. Shown is a maximum likelihood tree based on an alignment of DnaE (DNA polymerase III, α subunit). This tree strongly supports (97/100 bootstrap trees) the grouping of *Hodgkinia* within the Rhizobiales. Asterisks indicate 100% bootstrap support; values less than 70% are not shown. Scale bar denotes substitutions per site.

**Figure 6:**
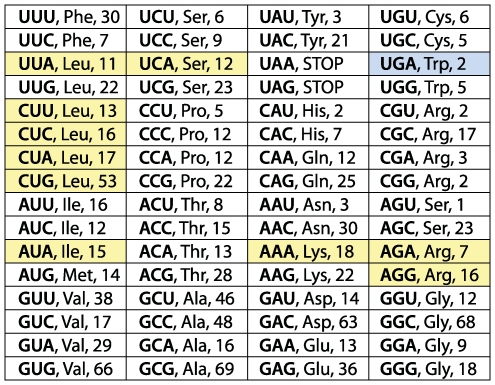
The count for all sense codons in the *Hodgkinia* genome covered by a peptide in the proteomic analysis. All sense codons were covered at least once. Codons in yellow are known to have undergone a recoding or been completely lost in other genomes but were shown here to be present and follow the universal code in *Hodgkinia*. The recoded UGA codon is colored in blue.

The long branch lengths for the *Hodgkinia* lineage in both rDNA and protein trees (Figure 4, Figure 5, and Figure S1) indicate a fast substitution rate, a situation typical of reduced bacterial genomes. Because the average percent identity of *Hodgkinia* proteins to their top hits in the GenBank non-redundant database was only 39.5%, it was difficult to rule out other recoding events based solely on sequence comparisons. To eliminate the possibility of other such changes in the genetic code, and to experimentally verify the UGA Stop→Trp recoding, shotgun protein sequencing by mass spectrometry [25] was used to sequence peptides derived from cicada bacteriomes. These peptide sequences ruled out any other codon reassignments, and experimentally confirmed the predicted UGA Stop→Trp code change (Figure 6 and Table S1).

**Figure 7:**
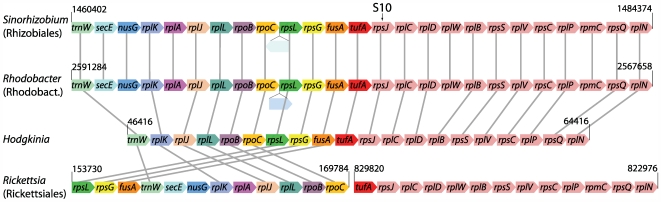
Gene order analysis shows that *Hodgkinia* is not within the Rickettsiales. Homologous individual genes in the *trnW*-*fusA* block (as ordered in *Hodgkinia*) are color-coded to highlight differences in gene order; genes in the *tufA*-*rplN* block (as ordered in *Hodgkinia*) are all colored pink as there are no gene order changes in this set of genes. Unrelated gene insertions are indicated with unlabeled lightly shaded boxes. Grey lines link up homologous genes. The S10 gene is indicated at the top of the figure. Genomic positions are indicated with black numbers; note that in Rickettsiales the *trnW*-*fusA* and *tufA*-*rplN* gene blocks are not contiguous on the genome. The gene order of *Hodgkinia* is compatible with the Rhizobiales and Rhodobacteraceae (with some gene loss in *Hodgkinia*), but not with Rickettsiales. Additional sequenced Rhizobiales (*Brucella melitensis* 16 M), Rhodobacteraceae (*Jannaschia* sp. CCS1) and Rickettsiales (*Wolbachia* endosymbiont of *Drosophila melanogaster*, *Ehrlichia canis* str. Jake, and *Anaplasma marginale* str. St. Maries) were examined; only one is depicted as the representative gene order for these groups.

Phylogenetic analysis of 16S rDNA sequences, including two newly acquired sequences from symbionts of other cicada species, shows that the cicada symbionts form a highly supported clade that falls within the α-Proteobacteria but outside of the Rickettsiales (Figure 4). The complete genome allowed additional phylogenetic analysis to further establish the placement of *Hodgkinia* within the α-Proteobacteria. Phylogenetic trees based on protein sequences (Figure 5 and Figure S1) support the grouping of *Hodgkinia* in the Rhizobiales, although the support was not always strong and trees made with some individual protein sequences placed it within the Rickettsiales with weak support (data not shown). We therefore looked for additional evidence in the form of gene order to further resolve the placement of *Hodgkinia*. The “S10” region (corresponding to the genomic region flanking ribosomal protein *rpsJ*) is a highly conserved cluster of genes that shares blocks of gene order conserved between Bacteria and Archaea [26]. The Rickettsiales have gene rearrangements and broken colinearity in this region that are unique within the α-Proteobacteria ([27] and Figure 7). *Hodgkinia* does not share these genomic signatures, instead showing perfect colinearity with genomes in the Rhizobiales and Rhodobacteraceae (Figure 7). These data rule out *Hodgkinia*'s grouping within the Rickettsiales, but do not entirely preclude a common ancestor with them, as *Hodgkinia* could have diverged from other Rickettsiales before the S10 region rearrangement.

The accurate placement of *Hodgkinia* within the α-Proteobacteria is confounded by both long branch attraction (LBA) and large differences in GC contents between different members of the α-Proteobacteria. LBA is expected to incorrectly associate *Hodgkinia* with the Rickettsiales, since these two lineages have the longest branches on the tree. Therefore, the fact that most analyses place *Hodgkinia* outside the Rickettsiales is significant. Conversely, the GC content bias is expected to incorrectly group sequences that are similar in GC content but that are not truly related by ancestry, and this artifact might tend to place *Hodgkinia* outside of the Rickettsiales, since *Hodgkinia* and most other non-Rickettsial α-Proteobacteria have high GC contents. We therefore tested all possible permutations in the placement of the *Hodgkinia* clade shown in Figure 4 under a model that does not assume nucleotide composition homogeneity among taxa [28],[29]. *Hodgkinia* did not group with the Rickettsiales in any of the highest scoring trees (Figure 4), suggesting that *Hodgkinia*'s grouping in the Rhizobiales was not a function of GC content bias. Overall, the results from the phylogenetics of proteins and 16S rDNA, as well as from gene order comparisons, strongly argue for the grouping of *Hodgkinia* with the Rhizobiales.

## Discussion

### Implications for the evolution of UGA Stop→Trp recoding events

All previously confirmed UGA Stop→Trp recoding events have occurred in genomes with low GC content: the mitochondria of Metazoa and Fungi, some Protist mitochondria, and certain bacteria in the Firmicutes [11]. (This same recoding may have occurred in the nuclear genomes of some Ciliates, but information on those genomes is limited [16]). Proposed evolutionary mechanisms for genetic code reassignments fall into three groups: the codon capture hypothesis [14],[15], involving the extinction and reassignment of codons; the genome reduction hypothesis, under which the pressure to minimize genome content drives the recoding of some codons, reducing the number of tRNAs [30]; and the ambiguous translation hypothesis, under which a single codon is temporarily read in two different ways, with a subsequent loss of the original meaning of the code [12],[31]. These hypotheses are not mutually exclusive and may apply more to some recoding events than to others [12]. For example, the pioneering ideas of Osawa and Jukes on this topic [14] involved loss of the corresponding tRNA following the extinction of a codon. Also, ambiguous translation, which is known for *Bacillus subtilis* [32], could facilitate a transition through the codon extinction route or the genome reduction route.

Codon capture requires the changing of one codon to another synonym though an initial codon extinction step potentially resulting from biases in nucleotide base composition. All previously described cases of UGA Stop→Trp recoding occur in GC-poor genomes, and this recoding has been proposed to result from genome-wide replacement of UGA by UAA, due to AT-biased mutational pressure [14],[15]. Under this explanation, the extinction of UGA Stop allows UGA to later reappear, recoded as an amino acid. Several arguments weigh against the codon capture hypothesis [11],[12]; most relevant is the fact that, in mitochondrial genomes, there is no association between the codons that undergo a reassignment and those that are expected to potentially disappear due to GC content bias [12]. Tallying stop codons in α-Proteobacteria with complete genomes also weighs against codon extinction as an initial step in this recoding event: although UGA codons are fewest in small and AT-biased genomes, in no case does UGA approach extinction. Among previously sequenced α-Proteobacteria (excluding *Hodgkinia*), even the smallest and most AT-biased genomes retain over 100 genes using UGA as Stop (e.g., there are 137 UGA Stop codons in the 1.11 Mb genome of *Rickettsia prowazekii*, which has a GC content of only 29%). In α-Proteobacteria with GC-rich genomes, UGA is the most frequent of the three stop codons and is typically used in a majority of genes (typically 50%–70% of coding genes end in UGA). Thus, the combination of phylogenetic evidence, which places *Hodgkinia* in the GC-rich Rhizobiales, and UGA usage patterns in extant α-Proteobacteria weigh strongly against UGA extinction as a causal step in the observed recoding.

**Figure 8:**
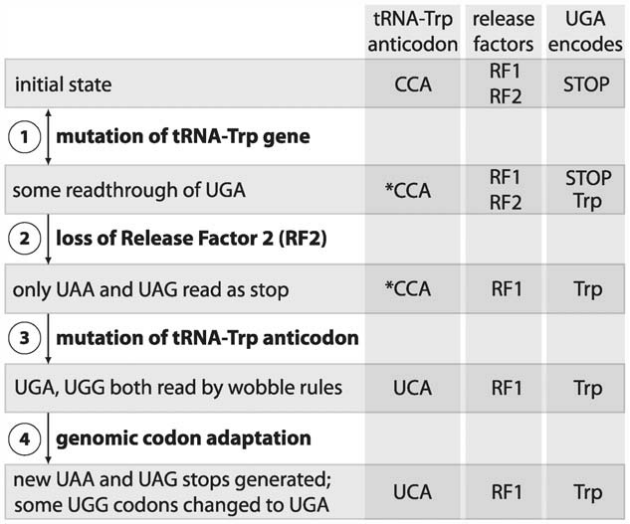
Model showing the mechanism of the UGA Stop→Trp recoding in the *Hodgkinia* genome. The asterisks refers to a tRNA that is identical in anticodon sequence to the canonical version but underwent a distal mutation which produced a structural change allowing A-C mismatches at the indicated position. Evidence suggesting that UGG codons are being changed to UGA codons comes from the *Hodgkinia* coding regions: of the 701 tryptophans in *Hodgkinia* proteins, almost half (48%) are coded by UGA.

We suggest an alternative hypothesis, implicating genome reduction as the primary driver of the UGA recoding, to explain the coding change observed in *Hodgkinia* (Figure 8). As in the ambiguous translation hypothesis, the recoding would first be enabled by the relaxed codon recognition of a mutated tRNA-Trp as promoted by structural changes in the tRNA [31] (Figure 8, step 1). For example, point mutations in either the D- or anticodon-arms of tRNA can induce C-A mispairing at the third codon position [33],[34]. In the presence of such alternative coding, RF2 is no longer essential and thus can be lost through the ongoing process of genome reduction (step 2). This is similar to the scenario envisioned in the codon capture hypothesis, except that in our case UGA does not need to have gone extinct before RF2 is lost. The further changes observed in *Hodgkinia* would evolve readily since they involve single base changes driven by positive selection; these include a change in the tRNA-Trp anticodon (step 3) and shifts in stop codon usage (step 4).

Since UGA Stop→Trp has evolved independently in other small genomes such as *Mycoplasma* and mitochondria, the case of *Hodgkinia* weighs in favor of genome reduction, and specifically loss of RF2, as the common force driving UGA Stop→Trp recoding events. Some of the Mollicutes, including *Mycoplasma*, and certain mitochondrial lineages are the other clear cases of this recoding event, and these genomes also have been characterized by a history of ongoing gene loss [22]. Of course, some small genomes do not show this recoding, and we do not expect the consequences of genome reduction to be predictable in each case. For example, the highly reduced genome of *Carsonella ruddii*, which retains UGA Stop and RF2, exhibits an unusual feature of having many overlapping genes with the most common overlap consisting of ATGA, in which ATG is the start of the downstream genes and TGA is the stop of the upstream gene [35], a situation that might act to conserve UGA Stop and RF2 in the genome.

At the initial loss of RF2, the additional C-terminal length imposed on UGA-ending proteins might be expected to impose some deleterious effects. It is possible that the functionality of proteins with such extensions could be enhanced in *Hodgkinia* due to an abundance of protein-folding chaperonins, similar to the high levels of GroEL seen in other symbiotic bacteria with small genomes [36],[37]. Indeed, analysis of the shotgun proteomic data for *Hodgkinia* shows that homologs of GroEL and DnaK are the two most abundant proteins in the cell (Table 2). Additionally, the shortened gene lengths observed in *Hodgkinia* relative to homologs in other genomes (Table 1) indicate that, if UGA-ending proteins were once extended due to recoding, they have since been reduced in length by the generation of new UAG and UAA stop codons. Other models are possible, such as the loss of RF2 effected by a change in the tRNA-Trp anticodon from CCA to UCA instead of distal mutations. Similarly, it is formally possible that *Hodgkinia* went through a period of AT bias under which the recoding occurred, with a subsequent shift to GC bias as is seen in the present genome. Because phylogenetic evidence favors placement of *Hodgkinia*'s in the Rhizobiales and not within any group characterized by AT rich genomes, we consider this scenario unlikely. Regardless of the recoding mechanism, however, this example provides a rare case in which the loss of an “essential” gene (RF2) in a highly reduced bacterial genome can be compensated by a few simple steps, namely the adaptive fixation of several point mutations.

**Table 2:** Homologs of the chaperones GroEL and DnaK are the most abundant proteins in *Hodgkinia*.

Gene	Pathway	Category	EmPAI	Num peptides
GroEL	Chaperonin Hsp60	Protein folding	2.62	18
DnaK	Chaperonin Hsp70	Protein folding	1.84	16
HisI	His synthesis	Amino acids	1.70	5
HisD	His synthesis	Amino acids	1.18	7
HCDSEM_115	Redox reactions	Unknown	1.02	5
CysK	Cys and Met synthesis	Amino acids	0.88	6
HisB	His synthesis	Amino acids	0.64	2
GlyA	Ser synthesis	Amino acids	0.47	5
HCDSEM_044	Phosphatase	Unknown	0.44	4
HisH	His synthesis	Amino acids	0.37	2
HisA	His synthesis	Amino acids	0.30	2
HCDSEM_125	Redox reactions	Unknown	0.23	2
CysI	Sulphur metabolism	Amino acids	0.22	3
TufA	EF-Tu	Translation	0.18	2
MetH	Met synthesis	Amino acids	0.09	3

The exponentially modified protein abundance index (emPAI) is a rough measure of relative protein amounts in complex mixtures, derived from the number of sequenced peptides and normalized by the expected number per protein [58]. All proteins from *Hodgkinia* with at least 2 unique peptides are ranked by their emPAI values. Based on homology of the 15 proteins identified in *Hodgkinia*, 60% (9/15) were involved in amino acid synthesis, 20% (3/15) could not be assigned to a general metabolic function, 13% (2/15) were involved in protein folding and stability, and 7% (1/15) were involved in translation. These results are not a complete listing of all expressed proteins, as exhaustive coverage of the symbiont proteome is difficult because the bacteria cannot be grown in pure culture, resulting in massive contamination from insect proteins. Therefore, even those proteins with only two mapped peptides may be abundant proteins in the cell.

### Unusual base composition in a reduced bacterial genome

The mechanisms that give rise to GC-content differences in bacterial genomes are unclear, although variations in the replication and/or repair pathways are often suggested as candidates [38]–[40]. Various lines of evidence support this idea, including a correlation between genome GC content and the types of DNA polymerase III, α subunit (DnaE) encoded in a genome [41] and the discovery of point mutations affecting the repair enzyme MutT that can detectably change the GC content of *Escherichia coli* [38]. One mechanistic clue is the correlation between genome size and GC content, a universal pattern in previously studied bacterial and archaeal genomes (Figure 1). Until now, this tendency has been especially pronounced in obligate intracellular bacterial genomes. Two (not necessarily mutually exclusive) hypotheses have been forwarded to explain this base composition bias in genomes of intracellular organisms. The first is an adaptive argument, based on selection for energy constraints [42]: synthesis of GTP and CTP require more metabolic energy, and ATP is the most common nucleotide in the cell because of its ubiquitous role in cellular processes. Therefore, competition for scarce metabolic resources has been hypothesized to force intracellular genomes to low GC values. The second hypothesis relates to mutational pressure resulting from altered capacity for DNA repair [43]. Small intracellular genomes typically lose many repair genes, and these organisms therefore are expected to be deficient in their ability to repair damage caused by spontaneous chemical changes. This is particularly expected in organisms such as endosymbionts in which genetic drift plays a major role in sequence evolution [43]. Indeed, recent experiments in *Salmonella* strongly support this hypothesis [44].

Our results weigh against the energetic hypothesis because *Sulcia*, living in the same bacteriome and presumably exposed to the same metabolite pool, has a GC content of 22.6% (J.P.M, B.R.M, and N.A.M., unpublished data), almost identical to the GC content of 22.4% for the previously published *Sulcia* genome from Glassy-winged sharpshooter [6]. One would expect that if the metabolite pool caused an increase in GC content in *Hodgkinia*, the same trend would be observed in *Sulcia*. Additionally, the GC content of the third position in 4-fold degenerate sites (which should be under little or no selective pressure) in the *Hodgkinia* genome is 62.5% (Table S2), consistent with mutational pressure as a cause of elevated genomic GC content.

Collectively, these data suggest that the replicative process or mutagenic environment of *Hodgkinia* differ from those of other small-genome α-Proteobacteria and other small genome insect symbionts. *Hodgkinia* has only two genes involved in replication (*dnaE*, DNA polymerase III, α subunit; and *dnaQ*, DNA polymerase III, ε subunit), implicating them as primary targets for future study of the source of GC bias. Regardless of the mechanisms involved in shifting genomic GC contents, our results indicate that low GC content is not an inevitable consequence of loss of repair enzymes, since *Hodgkinia* has no detectable repair enzymes (and is thus more extreme in this regard than previously sequenced symbiont genomes, which show partial loss of repair enzymes).

### *Candidatus* Hodgkinia cicadicola, a symbiont of cicadas

Our finding that two other cicada species contained symbionts belonging to the same clade, based on 16S rDNA genes (Figure 4) suggests that this symbiont infected an ancestor of cicadas and subsequently has been transmitted maternally, a typical history for bacteriome-dwelling insect symbionts [45],[46]. In such cases, the symbiont is restricted to its particular group of insect hosts, and restriction to cicada hosts is highly likely for this case. We propose the candidate name *Candidatus* Hodgkinia cicadicola for this α-Proteobacterial symbiont of cicadas, with the genus name referring to the biochemist Dorothy Crowfoot Hodgkin (1910–1994), and the species name referring to presence only in cicadas. Distinctive features include restriction to cicada bacteriomes, large tube-shaped cells, a high genomic GC content, a recoding of UGA Stop→Trp, and the unique 16S rDNA sequence ACGAGGGGAGCGAGTGTTGTTCG (positions 535–557, *E. coli* numbering).

## Materials and Methods

### Genome sequencing and annotation

Female cicadas were collected in and around Tucson, Arizona, USA. Tissue for genome sequencing was prepared from bacteriomes dissected in 95% ethanol and cleaned up in Qiagen's DNeasy Blood and Tissue Kit. DNA was prepared for the Roche/454 GS FLX pyrosequencer [47] following the manufacturer's protocols. Sequencing generated 523,979 reads totalling 116,176,938 bases, and these were assembled using the GS De novo Assembler (version 1.1.03) into 1029 contigs. Contigs expected to be from the *Hodgkinia* genome were identified by BLASTX [48] against the GenBank non-redundant database and the associated reads were extracted and reassembled to construct the *Hodgkinia* genome. Eleven contigs with an average depth of 73× were generated representing 143,582 nts of sequence with an average GC content of 58.4%. The order and orientation of the 11 contigs were predicted using the “.fm” and “.to” information appended to read names encoded in the 454Contigs.ace file and these joins were confirmed by PCR and Sanger sequencing.

Illumina/Solexa sequencing [49] generated 12,965,640 reads totalling 505,659,960 nts. These data were mapped to the *Hodgkinia* genome using MUMMER [50] (nucmer -b 10 -c 30 -g 2 -l 12; show-snps -rT -×30) to an average depth of 43×. Forty-five homopolymeric nucleotide runs were adjusted in length based on the Illumina data. Annotation was carried out as described previously [6], except that NCBI genetic code 4 (TGA encoding tryptophan) was used to computationally translate the predicted protein-coding genes. The *Candidatus* Hodgkinia cicadicola genome has been deposited in the GenBank database with accession number CP001226.

### Microscopy and 16S rDNA amplification

*D. semicincta* bacteriomes were dissected in PBS and gently disrupted with a mortar and pestle. Cells were fixed as described [51] and imaged on a Zeiss 510 Meta microscope. The probe sequences were Cy3-CCAATGTGGGGGWACGC (*Sulcia*) and Cy5-CCAATGTGGCTGACCGT (*Hodgkinia*). The scale bar in Figure 2 generated by the microscope software was overlaid with a plain white bar for legibility.

The PCR conditions used to amplify *Magicicada cassini* (Brood XIII, Chicago, Illinois) and *Diceroprocta swalei* (Tucson, Arizona) 16S rDNA were 94°C for 30 seconds followed by 35 cycles of 94°C 15 seconds, 58°C 30 seconds, 72°C 2 minutes using the primers 10F_ALPHA (AGTTTGATCCTGGCTCAGAACG) and 1507R (TACCTTGTTACGACTTCACCCCAG). Amplicons were cloned into Invitrogen's TOPO TA PCR2.1 kit and sequenced. The *D. swalei* and *M. cassini* 16S rDNA sequences have been deposited in the GenBank database with accession numbers FJ361199 and FJ361200, respectively.

### Phylogenetics

The initial assignment of the *Hodgkinia* 16S rRNA sequence was based on the Naive Bayesian classifier [19] at the Ribosomal Database Project (RDP) [20]; this uses a bootstrapping procedure involving resampling of sequence fragments with replacement and assignment of individual fragments to taxonomic units represented in this large database. The three *Hodgkinia* 16S rDNA sequences, sampled from bacteriomes of *D. semicincta* and two additional cicada species (*M. cassini* and *D. swalei*), were aligned to the Bacterial 16S rDNA model at the RDP, and the remaining sequences used in the generation of Figure 4 were also obtained from the RDP. The maximum likelihood tree in Figure 4 was generated using RAxML [52] under the GTRGAMMA model of sequence evolution. The clade consisting of the *Hodgkinia* sequences was moved to all other possible positions on the tree in Mesquite [53], and the log likelihood of each of these trees was estimated using the non-homogenous model implemented in nhPhyML [29] under a 4 category discrete gamma model using the shape parameter estimated from PUZZLE [54].

The protein sequence used in generating Figure 5 was DnaE (DNA polymerase III, α subunit), and the proteins used in generating Figure S1 were DnaE, InfB (translational initiation factor IF2), TufA (translational elongation factor Tu), RpoB (RNA polymerase, β subunit), and RpoC (RNA polymerase, β′ subunit). Individual alignments for each gene were generated using the linsi module of MAFFT [55] and (in the 5-protein alignment) concatenated. Columns containing gap characters were removed, leaving 861 columns in the DnaE alignment and 4152 columns in the 5-protein alignment. Parameters for a 1 invariant/4 Gamma distributed rate heterogeneity model were estimated using PUZZLE, and maximum likelihood trees were computed with PROML from the PHYLIP package [56] using the JTT model of sequence evolution. One hundred bootstrap datasets were generated using SEQBOOT from PHYLIP, trees were calculated as above, and bootstrap values for these trees were mapped back on the maximum likelihood tree calculated from PROML using RAxML. The family and order names and groupings on Figure 4, Figure 5, and Figure S1 were taken from [57] and the RDP website [20]. The genomes used in the phylogenetic analysis were (the accession numbers noted with asterisks were used in generating Figure 7): *Zymomonas mobilis* subsp. mobilis ZM4 (NC_006526), *Erythrobacter litoralis* HTCC2594 (NC_007722), *Novosphingobium aromaticivorans* DSM 12444 (NC_007794), *Sphingopyxis alaskensis* RB2256 (NC_008048), *Candidatus* Pelagibacter ubique HTCC1062 (NC_007205), *Rickettsia rickettsii* str. Iowa (NC_010263), *Rickettsia typhi* str. Wilmington (NC_006142*), *Neorickettsia sennetsu* str. Miyayama (NC_007798), *Wolbachia pipientis* (NC_010981), *Wolbachia* endosymbiont of *Drosophila melanogaster* (NC_002978), *Anaplasma phagocytophilum* HZ (NC_007797), *Anaplasma marginale* str. St. Maries (NC_004842), *Ehrlichia ruminantium* str. Gardel (NC_006831), *Ehrlichia canis* str. Jake (NC_007354), *Rhodospirillum rubrum* ATCC 11170 (NC_007643), *Magnetospirillum magneticum* AMB-1 (NC_007626), *Acidiphilium cryptum* JF-5 (NC_009484), *Gluconobacter oxydans* 621H (NC_006677), *Gluconacetobacter diazotrophicus* PAl 5 (NC_010125), *Paracoccus denitrificans* PD1222 (NC_008686/NC_008687), *Rhodobacter sphaeroides* ATCC 17025 (NC_009428*), *Jannaschia* sp. CCS1 (NC_007802), *Silicibacter pomeroyi* DSS-3 (NC_003911), *Silicibacter* sp. TM1040 (NC_008044), *Roseobacter denitrificans* OCh 114 (NC_008209), *Caulobacter crescentus* CB15 (NC_002696), *Caulobacter* sp. K31 (NC_010338), *Maricaulis maris* MCS10 (NC_008347), *Brucella melitensis* 16M (NC_003317/NC_003318), *Brucella abortus* S19 (NC_010742/NC_010740), *Bartonella bacilliformis* KC583 (NC_008783), *Bartonella henselae* str. Houston-1 (NC_005956), *Mesorhizobium loti* MAFF303099 (NC_002678), *Mesorhizobium* sp. BNC1 (NC_008254), *Agrobacterium tumefaciens* str. C58 (NC_003062/NC_003062), *Rhizobium etli* CFN 42 (NC_007761), *Sinorhizobium medicae* WSM419 (NC_009636), *Sinorhizobium meliloti* 1021 (NC_003047*), *Rhodopseudomonas palustris* BisA53 (NC_008435), *Rhodopseudomonas palustris* BisB18 (NC_007925), *Bradyrhizobium japonicum* USDA 110 (NC_004463), *Bradyrhizobium* sp. BTAi1 (NC_009485), *Nitrobacter hamburgensis* X14 (NC_007964), *Nitrobacter winogradskyi* Nb-255 (NC_007406), *Xanthobacter autotrophicus* Py2 (NC_009720), and *Escherichia coli* str. K12 substr. MG1655 (NC_000913).

### Proteomics

Total protein was prepared from the bacteriomes of 10 female *D. semicincta* by homogenizing in 4 ml Buffer H (2% SDS, 100 mM Tris, 2% β-mercaptoethanol, pH 7.5) followed by centrifugation at 100,000×g for 30 min. The supernatant was recovered and precipitated in 12% TCA followed by 3 washes in cold acetone. The resulting protein pellet was resuspended in 150 µl sample loading buffer, and 30 µl (∼60 µg) of this sample was loaded onto a well of a 11 cm×8 cm×1.5 mm 10% acryl amide gel. Electrophoresis was performed in a mini cell (Bio-Rad) at 130 V. The entire lane was cut into 12 sections, and proteins in each section were identified by LC-MS/MS analysis.

The gel bands were washed, homogenized, reduced, alkylated and subjected to overnight in-gel tryptic digests. The peptide mixture was extracted, dried in speed-vac and dissolved in a 15 µl of 5% formic acid. The LC-MS/MS experiments were performed on a Q-TOF 2 mass spectrometer equipped with the CapLC system (Waters Corp., Milford, MA). The stream select module was configured with a 180 µm ID×50 mm trap column packed in-house with 10 µm R2 resin (Applied Biosystems, Foster City, CA) connected in series with a 100 µm ID×150 mm capillary column packed with 5 µm C18 particles (Michrom Bioresources, Auburn, CA) using a pressure cell. Peptide mixtures (10 µl) were injected onto the trap column at 9 µl/min and desalted for 6 min before being flushed to the capillary column. The peptides were then eluted from the column by the application of a series of mobile phase B gradients (5 to 10% B in 4 min, 10 to 30% B in 61 min, 30 to 85% B in 5 min, 85% B for 5 min). The final flow rate was 250 nl/min. Mobile phase A consisted of 0.1% formic acid, 3% acetonitrile and 0.01% TFA, whereas mobile phase B consisted of 0.075% formic acid, 0.0075% TFA in an 85/10/5 acetonitrile/isopropanol/water solution. The mass spectrometer was operated in a data dependent acquisition mode whereby, following the interrogation of MS data, ions were selected for MS/MS analysis based on their intensity and charge state +2, +3, and +4. Collision energies were chosen automatically based on the m/z and charge-state of the selected precursor ions. The MS survey was from m/z 400–1600 with an acquisition time of 1 sec whereas the trigged data-dependent MS/MS fragmentation scan was from m/z 100–2000 with an acquisition time of 2.4 sec.

The peak list was created using the Mascot distiller 2.2 software from Matrix Science (London, UK) using the default settings for Waters. The Mascot 2.2 search engine was used to assist in the search of the combined tandem mass spectra against a custom protein database. The custom protein database consisted of the *Hodgkinia* proteome, the nearly complete proteome of *Sulcia muelleri* from *Diceroprocta semicincta* (J.P.M., B.R.M., and N.A.M., unpublished), and the complete proteome from the pea aphid *Acyrthosiphon pisum* (build 1.1), the most closely related insect to *D. semicincta* for which a complete genome is available. The database contained 5,508,819 amino acids residues in 10,887 protein sequences. The parameters used for the searches were as follows: trypsin-specificity restriction with 2 missing cleavage site and variable modifications including oxidation (M), deamidation (N,Q), and alkylation (C). Both MS and MS/MS mass tolerance was set to 0.3 Da for the searching.

The Mascot significance threshold was set to 0.05, using MudPIT scoring, with a Mowse ion score cutoff of >31 (the cutoff for a peptide suggesting identity or extensive homology). The sequences in the custom proteome database were reversed to generate a decoy database for calculation of a false discovery rate, which was 2.6% (15 peptides found in the decoy database vs. 576 peptides found in the real database). For a peptide to be considered in the calculation of codon coverage (Figure 6), it had to originate from a protein with at least one other high-quality matching peptide. Eighty-seven (87) such peptides from 16 *Hodgkinia* proteins were found (Table S1). These peptides cover all 62 non-stop codons at least once; the peptides LIWPSAVLQAEEVWAGAR from HCDSEM_044 and VSCLIWTDINR from HisA span recoded UGA codons.

## Supporting Information

Figure S1 Phylogenetic trees made from concatenated protein alignments support *Hodgkinia* grouping with the Rhizobiales. The maximum likelihood tree is calculated from a concatenated alignment of DnaE (DNA polymerase III, α subunit), InfB (translational initiation factor IF2), TufA (translational elongation factor Tu), RpoB (RNA polymerase, β subunit), and RpoC (RNA polymerase, β′ subunit). Eighty-one of 100 bootstrap trees support the grouping. Scale bar denotes substitutions per site.
(0.22 MB PDF)

Table S1 High-quality peptides found in the proteomic analysis.
(0.47 MB PDF)

Table S2 Counts for the third position nucleotide in 4-fold degenerate family box codons. The overall GC content of the *Hodgkinia* genome is 58.4%, but the GC content of the third position of the family box codons is 62.5%, indicating a GC mutational bias. Note that in third positions following a C or T, there is a bias towards G over C (71.2% G vs. 28.8% C) but that the bias is switched in third positions following a G (22.4% G vs. 77.6% C).
(0.10 MB PDF)
