# Supplementary figures and images for: Origin of an Alternative Genetic Code in the Extremely Small and GC–Rich Genome of a Bacterial Symbiont
Source: PLoS Genet. 2009 Jul 17;5(7):e1000565. doi: 10.1371/journal.pgen.1000565 (PMC2704378; doi:10.1371/journal.pgen.1000565)

Figure S1

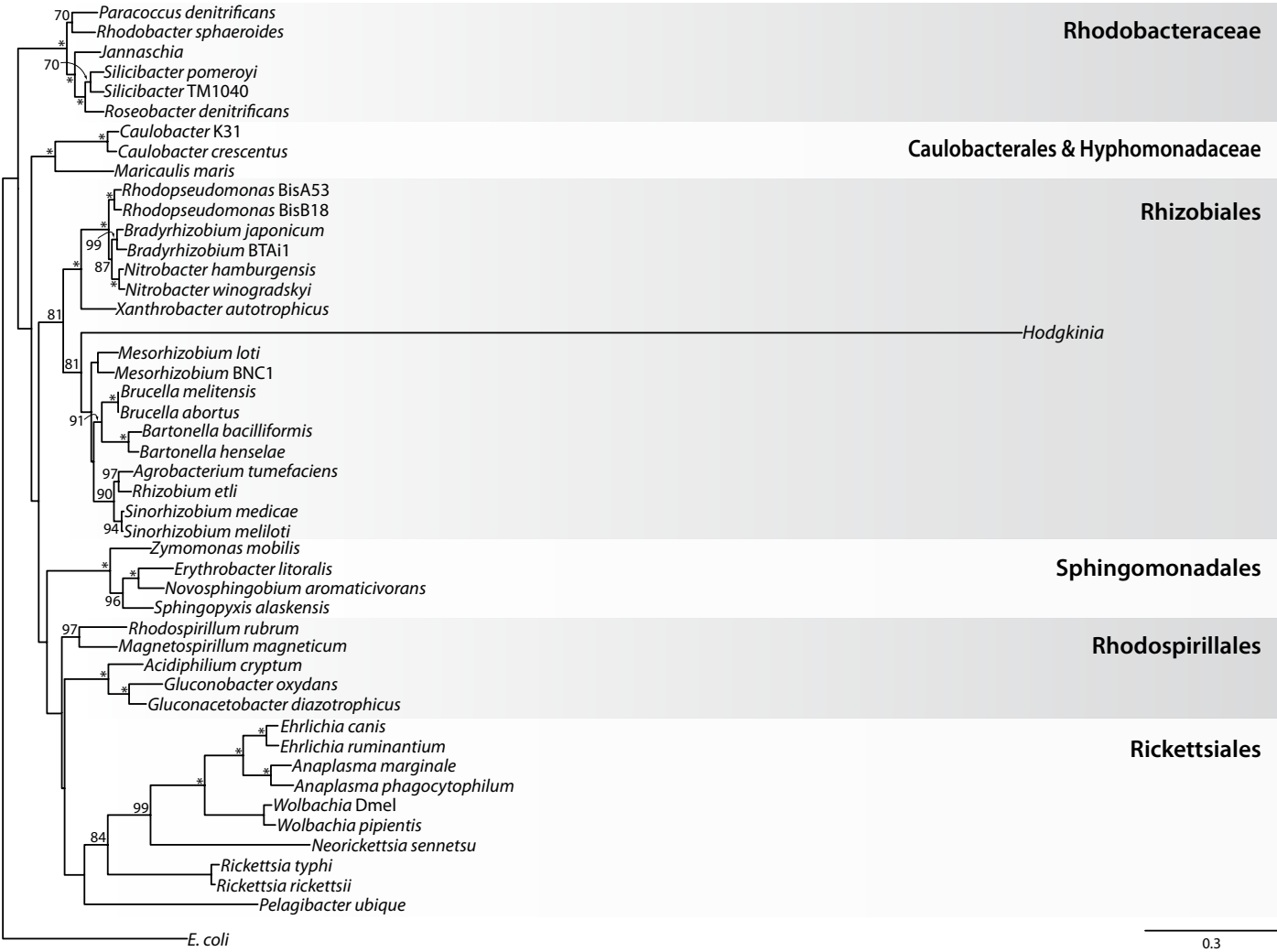

Supplement: Figure S1 — Phylogenetic trees made from concatenated protein alignments support Hodgkinia grouping with the Rhizobiales. The maximum likelihood tree is calculated from a concatenated alignment of DnaE (DNA polymerase III, α subunit), InfB (translational initiation factor IF2), TufA (translational elongation factor Tu), RpoB (RNA polymerase, β subunit), and RpoC (RNA polymerase, β′ subunit). Eighty-one of 100 bootstrap trees support the grouping. Scale bar denotes substitutions per site. (0.22 MB PDF) [file pgen.1000565.s001.pdf]
