# Supplementary material for: Origin of an Alternative Genetic Code in the Extremely Small and GC–Rich Genome of a Bacterial Symbiont
Source: PLoS Genet. 2009 Jul 17;5(7):e1000565. doi: 10.1371/journal.pgen.1000565 (PMC2704378; doi:10.1371/journal.pgen.1000565)
Supplement: Table S1 — High-quality peptides found in the proteomic analysis. (0.47 MB PDF) [file pgen.1000565.s002.pdf]

Table S1

GroL	L E N V T L D K<br>ctt gaa aac gtc acg tta gac aag<br>V G F D I L N R<br>gta ggc ttc gac att tta aac agg<br>V E D A L N A T R<br>gtt gaa gac gcg ctc aac gcc acg cgc<br>V G G V T E A E V K<br>gtt ggt ggc gtc acc gaa gct gaa gtt aag<br>Q L L F G V D A R<br>cag ctt ttg ttt ggc gtc gac gcc aga<br>G I D L A V D E V A K<br>ggt att gat ttg gca gtc gac gag gtg gcc aag<br>D T T T I V D G G G S K<br>gac acc aca acc atc gtt gac ggc ggg ggc agt aag<br>A V A T G A N P V D L K<br>gct gtg gca act ggc gcc aac ccc gta gac ctt aaa<br>A M A P P A V V G D E R<br>gct atg gcg ccg ccg gct gtt gtc gga gac gag cgc<br>D R V E D A L N A T R<br>gac agg gtt gaa gac gcg ctc aac gcc acg cgc<br>V G G D G V I T V E E A R<br>gtc ggg ggc gac ggc gtc ata acg gtt gaa gag gca agg<br>Q I G L D I A E A M Q R<br>cag att ggg cta gat att gct gaa gcg atg caa cgg<br>A Q W Q D S A S D Y E R<br>gcc cag tgg cag gac agc gcg tcc gac tac gag agg<br>Y G D M L A M G I V D P V K<br>tac ggc gac atg ctt gct atg ggc att gtg gac cca gtt aag<br>V L E S G S S S F G Y D A Q A G R<br>gtg cta gag tcc ggc tcg tcg tcg ttt ggt tat gat gcg caa gcc ggc cgc<br>M V C E L D N P Y V L L Y D R<br>atg gtg tgc gag ctg gac aac ccc tac gtg ctg ctg tac gac aga<br>E I E L G D K F E N V G A Q M V R<br>gaa ata gag ctg ggg gac aag ttt gaa aac gtc gga gct caa atg gtt cga<br>I S S L Q A L V P L L E T V V Q T N R<br>att tca agc ttg cag gcg ctc gtg ccc ctg cta gag acg gtg gtg cag acc aac agg
DnaK	V V D F L A S Q F K<br>gta gtc gac ttc ctg gcg tca cag ttt aaa<br>R Y D D E L V Q R<br>cga tac gac gac gag ctg gtt cag cgc<br>D A G T I A G L E V L R<br>gac gcg ggc aca ata gcg ggc ctc gag gtg ctg aga<br>L E A L V D D L I Q K<br>ctt gaa gcg tta gta gac gac cta att cag aaa<br>N Q A E S L V Y S A E K<br>aac caa gct gaa agc ctg gtg tat tcg gcg gaa aaa<br>T T P S V V A I T E A G D K<br>acg acg ccg tcc gta gta gca ata acc gaa gct ggc gac aag<br>A Y S P A Q V S A M V L S K<br>gct tac tcg ccg gcg cag gtg tcg gcg atg gtg ctg tca aaa<br>A K L E A L V D D L I Q K<br>gct aaa ctt gaa gcg tta gta gac gac cta att cag aaa<br>Q A V T N P H N T F Y A V K<br>caa gct gta acc aac cca cac aac acg ttc tac gcc gtt aag<br>I I N E P T A A A L A Y G L E K<br>ata ata aac gag cca acg gcg gca gcg ctg gcg tac ggg cta gag aag<br>S T N G D T F L G G E D F D A R<br>tca aca aac ggc gac acg ttt tta gga ggt gaa gac ttc gac gcc aga<br>S Q V F S T A E D G Q S A V T I K<br>tcg cag gtg ttt tca acg gca gaa gac ggc cag tca gcg gta aca ata aag<br>V I G I D L G T T N S C V A I A E G K<br>gta ata ggc att gat ttg ggc acg acc aac tcg tgc gta gca ata gcc gaa ggc aag<br>G V N P D E V V A V G A A I Q A G V L Q G D V K<br>ggt gtg aat ccc gac gaa gtt gta gcc gtg ggc gca gcc ata caa gcc ggc gtg ctc caa ggc gac gta aaa<br>V E L S S A P T T E V N L P F I T A S Q A G A K<br>gtg gag ctg tcg tca gct cca acc aca gaa gtg aac ttg ccg ttt att act gcg tct caa gcc ggc gcc aag<br>A T A E A H L G E A V T Q A V I T V P A Y F S D<br>A Q R<br>gcc aca gcc gaa gcc cat ctc ggg gag gcg gtg acg caa gcc gta atc acg gtg ccg gcg tac ttt agc gac<br>gcc cag cgc

HisD	S D S P G F G E F V K<br>tca gac tcg cct ggt ttt ggc gag ttt gtt aaa<br>T Y V T W L P T A R<br>acg tac gtc act tgg ctg ccg act gcg cgc<br>I V G P G N A F V A A K<br>att gtg ggg ccg ggc aac gcg ttt gtg gct gcg gcc aaa<br>F A S G L S V L D F M K<br>ttt gcg tcc ggg ctg tcg gtg ctg gac ttt atg aag<br>F A P D I D L E T V D V K<br>ttt gcg ccc gac atc gat ctg gag act gtg gac gtg aaa<br>P M L A L A P A C A A C A S A E G L S C H A L A<br>S A S R<br>cca atg ctg gcg ctg gcg cca gcg tgc gct gcg tgc gcg tcc gct gaa ggc ttg tcg tgt cac gct ctg gca<br>tcg gct agc agg<br>L T S A G A V F L G G G S P I V V G D Y V G G T<br>N H T L P T A S G A K<br>ctt aca tcg gct ggt gct gtg ttt cta ggt ggc ggc tct ccg att gtg gtc gga gac tac gtt ggg ggc aca<br>aac cac acg ctg ccc acc gcg tct ggc gcc aag
HCDSEM_115	Y A A E L Q Y L K<br>tat gcg gca gag ctt cag tac ctc aaa<br>V L E V T H Y T N R<br>gtg cta gaa gtc acc cac tac acc aac agg<br>I A Q L A Q Q P E V R<br>ata gcg cag cta gcg cag cag ccc gag gtg agg<br>V P N G P L T S Y L Q K<br>gtg cca aac ggg ccg ctg act tcg tac ctg caa aaa<br>L I A S G V L A A D L G G E P L N A D D R<br>ctg atc gcc tcc ggc gtg cta gcc gca gac ctc ggg ggt gag ccg ctg aac gcg gac gac cgc
HisI	M F A E A A S E Q R<br>atg ttt gct gag gcg gcg tcc gag cag cgt<br>V A V S A H D A T V S R<br>gtt gcg gta agc gcg cat gac gcc acc gtt tca aga<br>M L A E G L I Q I L K<br>atg ctt gct gaa ggc ttg att cag atc ttg aaa<br>Y A I K P V A G V G G L E L K<br>tac gct atc aag cca gtg gct gga gtt ggc ggc ttg gag ctg aaa<br>N A G V A A G G L L D S T A V L I A S A F N K<br>aac gcc ggt gtt gcc gct ggc ggg ctg ctc gat agc aca gcc gtg ctg att gcg agc gcg ttt aac aag
GlyA	A V A L L E A S A P S F R<br>gct gtg gcg ctg cta gaa gcc agc gcg ccc tcg ttc agg<br>V L Q L A D A F P V L Y K<br>gtt ttg cag ctg gct gac gcg ttt cct gtg ctt tac aaa<br>L V T G G T D T H V V L L D L R<br>ctg gtg act ggg ggc acc gac acc cac gtc gtt ctg ctc gac ctg agg<br>N V L P F D A L P S V V A S G L R<br>aac gtt ttg cca ttc gac gcc ttg cct tcg gtc gtg gcc tcg ggc tta agg<br>A V A Y G V D P I T G L V D M N S V L D L A I R<br>gct gtg gcc tac ggc gta gat ccg atc acg ggc ctc gtt gac atg aac agc gtg ctg gat ttg gcc ata cgc
CysK	L E S V N P L G S V K<br>ctc gag tcc gtc aac ccg ttg ggg tct gta aaa<br>T V L V I L P S L A E R<br>aca gtt tta gta ata ttg ccg tcg cta gcc gag cgc<br>G A L A S I A S A V G S T P V L R<br>gga gcg ctc gct tcg ata gct agc gcc gtt ggc agc acg cca gtg cta agg<br>G L S C I V V V P E G A S A E R<br>ggg ctc agc tgc atc gtg gtg gta ccg gag ggc gcg tca gcc gag agg<br>I E G S A L A N Q F E S P A N S K<br>atc gaa ggc tca gct tta gct aat cag ttt gaa agc cca gcc aac agc aaa<br>V D Y L V A G V G T G G T I T G V G E A L K<br>gta gac tac ctg gta gct ggc gtg ggc aca ggc ggc acg ata acg gga gta ggc gag gca cta aag

CysI	V G V V C G V L C A R<br>gtg ggc gtg gtg tgt ggc gtg ctg tgt gcg cgc<br>V L G L F H D V G V R<br>gtg cta ggc ctg ttt cac gac gtt ggc gtg agg<br>A P P G D V T A G D V E A L A T A A Q R<br>gcg ccg ccg ggc gac gtg acg gct ggc gac gta gaa gcg ctg gcg acg gca gcg cag cgc
HisB	F A S A A V P M D E A L A R<br>ttt gct agc gcg gct gtg ccc atg gac gag gcg tta gcg cgc<br>F E A G A A P A V E L A L V M L D A L A R<br>ttt gaa gct ggc gca gcg cca gcc gtt gag ctg gcg ctt gta atg tta gac gct ttg gct agg
HCDSEM_125	S G A V S L A T A P V V V A G G R<br>tct gga gct gtg tcg ttg gcc acg gcc ccc gtg gtg gtt gcc ggc ggc cgc<br>S F A T A E A F D Q H V V V L A S K<br>agc ttt gct acg gcg gaa gca ttc gac cag cac gtg gtg gtg ctg gct tct aag
HCDSEM_044	L V N A L L D A L S R<br>ctg gtt aac gcg ctg ctg gac gcg cta tcg cgt<br>A F V C G S P V W G R<br>gcg ttt gta tgt ggc tcg ccc gtt tgg ggg cga<br>F D V V A E A S A F N A L K<br>ttt gat gta gtt gct gaa gcc agc gcg ttt aac gcg ctc aag<br>L I W P S A V L Q A E E V W A G A R<br>ctg atc tgg ccg tca gcc gtg ctg caa gcc gag gaa gtt tga gct ggc gcg cgc
MetH	F L S L L A A E P D L A K<br>ttt tta agc ttg ctg gcg gct gag ccg gat ttg gcc aag<br>G V T T P L L V G G A T T S K<br>ggc gtc acg acg ccg ctg ctg gtg ggg gga gcc aca acc agc aag<br>S V T P E E V I G A F A S A G V P R<br>agc gta aca ccg gaa gaa gtg att ggc gcg ttt gct tcc gcc ggc gtg ccc cgg
HisA	V S C L I W T D I N R<br>gtc agc tgt cta atc tga act gat att aac aga<br>D G T L Q G V N F D A L D R<br>gat ggc acg ttg caa ggt gtt aat ttt gac gcg ctt gac cgc
TufA	E L L A A Y G Y L P D L T P I V R<br>gag ctg ctg gcg gcc tac ggc tac ttg ccg gac ctc acg cct atc gtt agg<br>V D Q V D D D E L V E L V E L E L R<br>gta gat cag gtg gac gat gac gag ctg gtt gag ctg gtg gag ctg gag ctt aga
HisH	V A V V D Y G V G N L K<br>gta gca gtt gtt gac tac ggg gtt ggc aac ttg aag<br>V V L P G V G S F G G C W R<br>gtt gtg ctg cct ggt gtg ggc agc ttt ggc ggc tgt tgg cgc
