# Supplementary material for: Origin of an Alternative Genetic Code in the Extremely Small and GC–Rich Genome of a Bacterial Symbiont
Source: PLoS Genet. 2009 Jul 17;5(7):e1000565. doi: 10.1371/journal.pgen.1000565 (PMC2704378; doi:10.1371/journal.pgen.1000565)
Supplement: Table S2 — Counts for the third position nucleotide in 4-fold degenerate family box codons. The overall GC content of the Hodgkinia genome is 58.4%, but the GC content of the third position of the family box codons is 62.5%, indicating a GC mutational bias. Note that in third positions following a C or T, there is a bias towards G over C (71.2% G vs. 28.8% C) but that the bias is switched in third positions following a G (22.4% G vs. 77.6% C). (0.10 MB PDF) [file pgen.1000565.s003.pdf]

Table S2

4 box codon	G	C	A	T	Codon total
Ala (GCN)	2511	1391	652	2034	6588
Gly (GGN)	614	1888	254	481	3237
Leu (CTN)	1755	453	849	911	3968
Pro (CCN)	449	375	326	346	1496
Arg (CGN)	323	1364	324	282	2293
Ser (TCN)	777	226	352	316	1671
Thr (ACN)	816	373	343	309	1841
Val (GTN)	2064	574	522	1280	4440
Total	9309	6644	3622	5959	25534
Percentage	36.5	26.0	14.2	23.3	
